# Supplementary material for: A structural and dynamic model for the assembly of Replication Protein A on single-stranded DNA
Source: Nat Commun. 2018 Dec 21;9:5447. doi: 10.1038/s41467-018-07883-7 (PMC6303327; doi:10.1038/s41467-018-07883-7)
Supplement: Supplementary file 1 — Supplementary Information [file 41467_2018_7883_MOESM1_ESM.pdf]

Supplementary Information

A structural and dynamic model for the assembly of Replication Protein A on single-stranded DNA

Yates et al,

Supplementary Figure 1

a

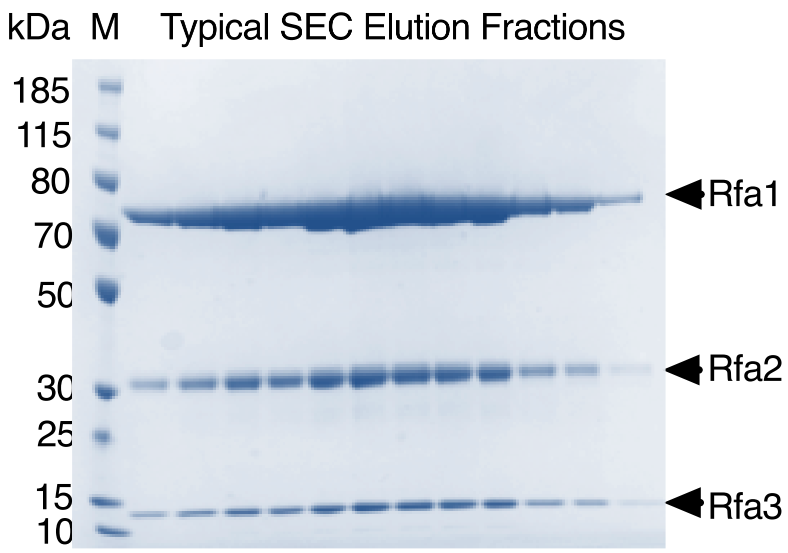

b

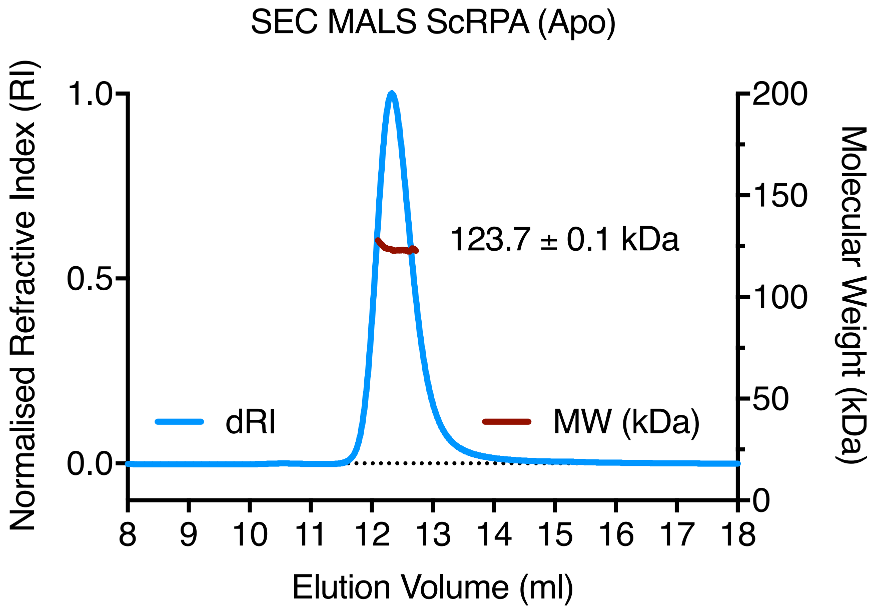

Supplementary Figure 1 – ScRPA Purification quality control
(a) Representative SDS-PAGE of purified RPA from gel filtration.
(b) Size exclusion chromatography coupled with multi-angle laser light scattering (SEC-MALLS) of Apo RPA suggesting a homogenous purified sample. The estimated MW of the RPA heterotrimer is ~117 kDa.

Supplementary Figure 2

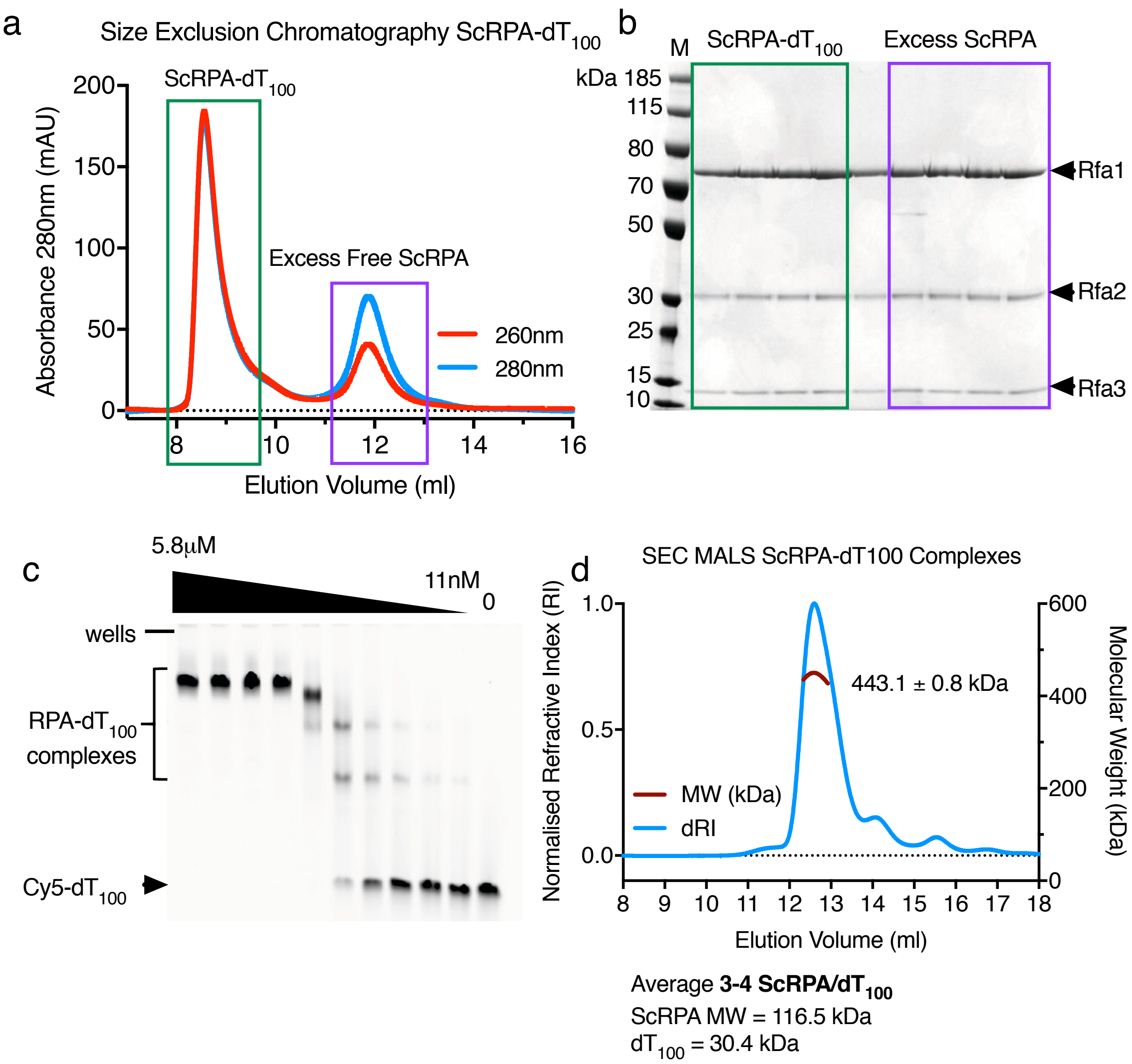

Supplementary Figure 2 – ScRPA-dT$_{100}$ purification for cryo-EM and stoichiometry assessment

(a) Size Exclusion Chromatography (SEC) of a ssDNA oligonucleotide (dT$_{100}$) saturated with ScRPA used for cryo-EM. Two wavelengths (260nm and 280nm) are monitored to ensure protein and nucleoprotein complexes. Elution peaks are highlighted by colored boxes and correspond to SDS-PAGE lanes in (b).

(b) SDS-PAGE of ScRPA-dT$_{100}$ nucleoprotein complexes and excess RPA purified by gel filtration (a).

(c) Electrophoretic mobility shift assays (EMSA) of increasing concentrations of ScRPA against Cy5-labelled dT$_{100.}$ Free DNA and Protein-DNA complexes were separated on a 5% Polyacrylamide Tris-Borate gel and show up 3 separable RPA-DNA species.

(d) Size exclusion chromatography coupled with multi-angle laser light scattering (SEC-MALLS) of ScRPA-dT$_{100}$ complexes. MALLS provide an

approximate molecular weight of 443 ± 6.5 kDa, which indicates 3-4 RPA molecules (116.5kDa per RPA) on a dT_{100} (30.4kDa).

Supplementary Figure 3

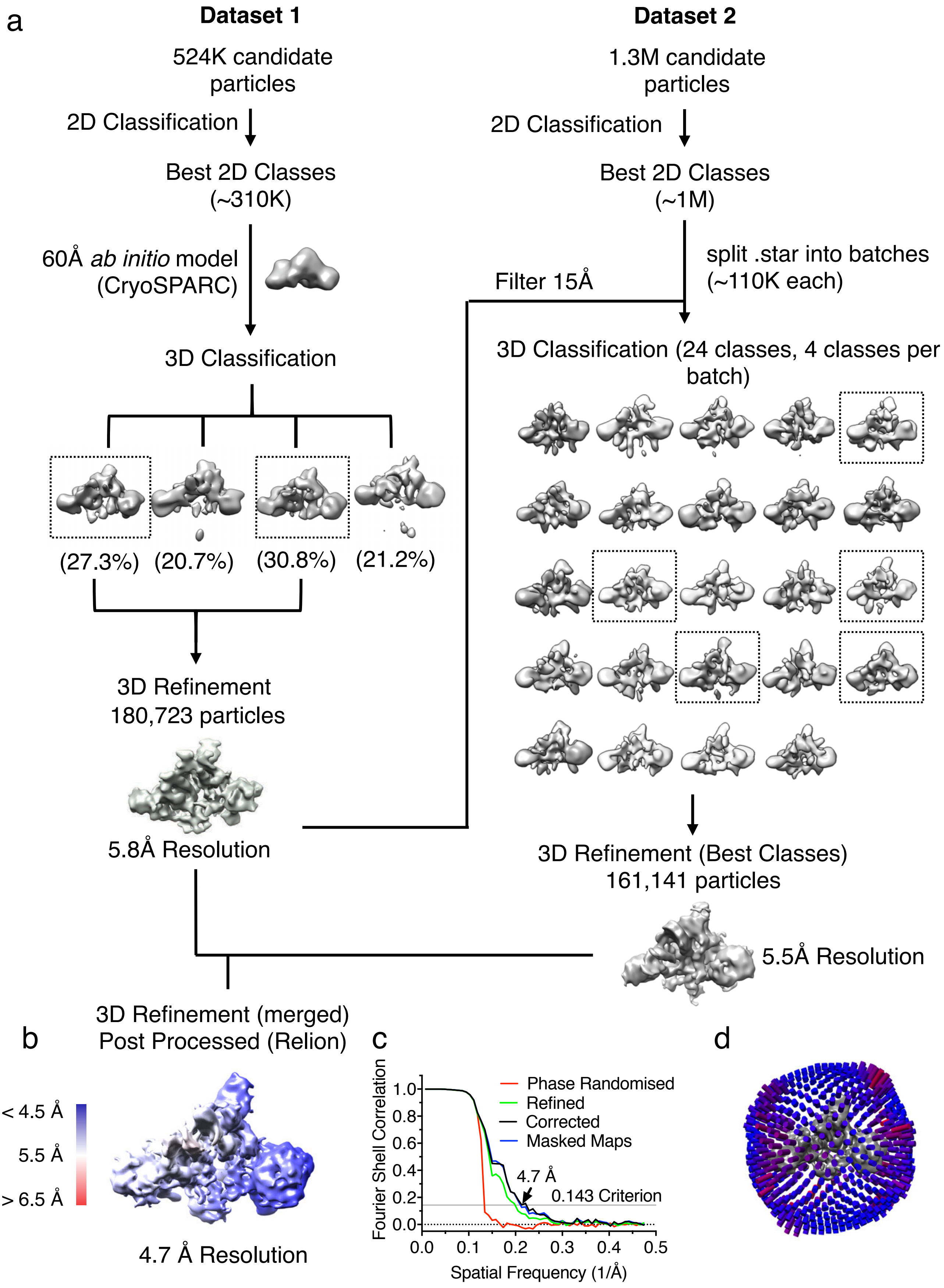

Supplementary Figure 3 – Reconstruction schematic corresponding to the Tri-C

(a) 3D reconstruction schematic for ScRPA trimerisation core showing processing of candidate particles that yielded a final 3D reconstruction.

(b) Post-processed final Tri-C reconstruction, colored by local resolution, calculated in Relion2.0,

(c) Estimated global resolution of 4.7 Å according to gold-standard Fourier shell correlation (FSC) calculated in Relion2.0.

(d) Angular distribution of the reconstruction.

Supplementary Figure 4

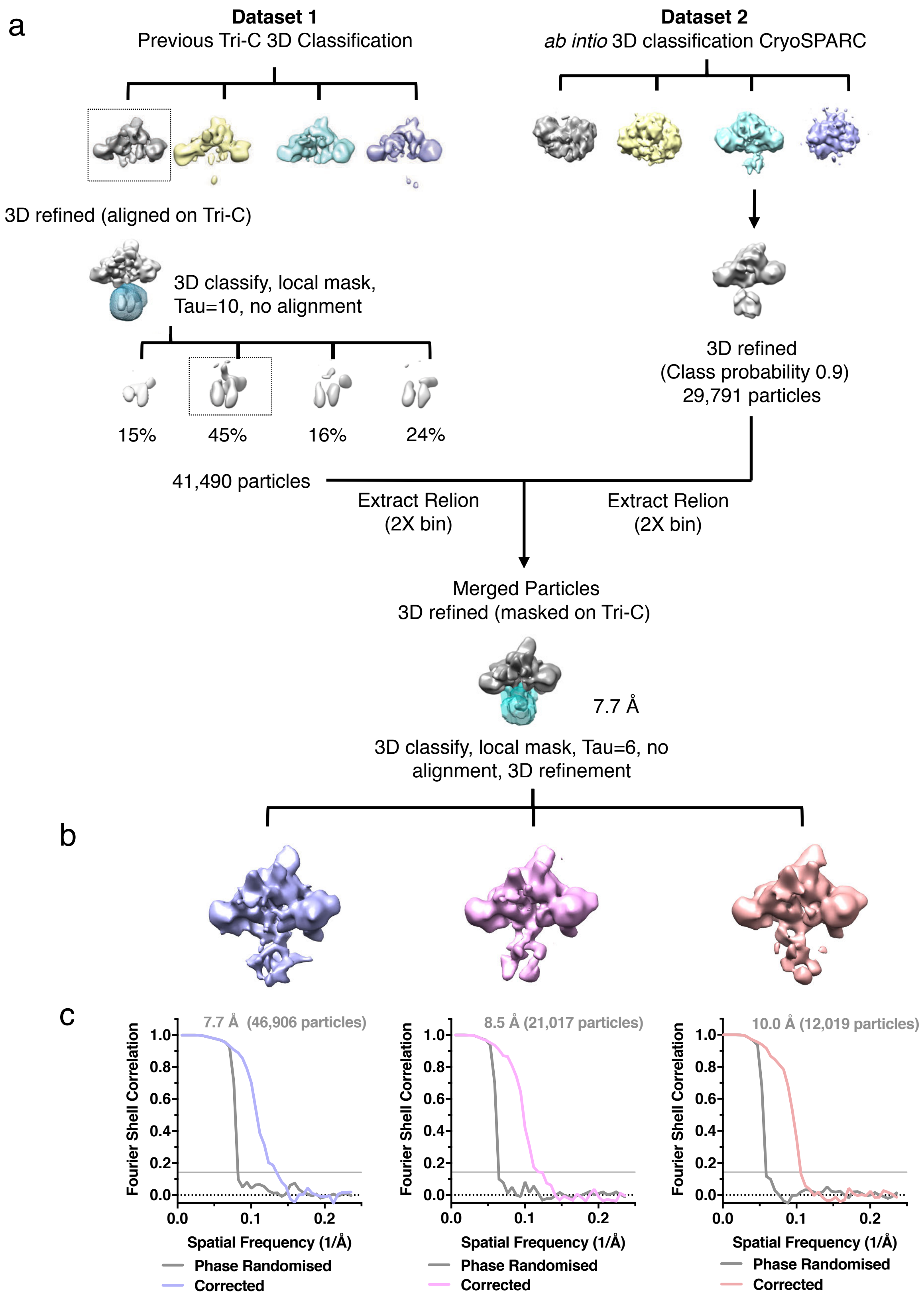

Supplementary Figure 4 – Reconstruction scheme for additional DBD

(a) 3D reconstruction processing schematic for ScRPA trimerisation core with an additional DNA-binding domain. Particles from dataset 1 and dataset 2, with evidence of an additional domain, were extracted and combined and twice binned to maximize the signal of this small domain (~10kDa). Particles were aligned on the Tri-C via masked a 3D refinement and sorted by 3D classification using a local mask. Images were binned twice to increase the signal of the small domain.

(b) Refinements of individual classes from 3D classification each post processed in Relion2.0 with (c) corresponding FSC curves showing numbers of particles and final resolution according to gold-standard Fourier shell correlation (FSC).

Supplementary Figure 5

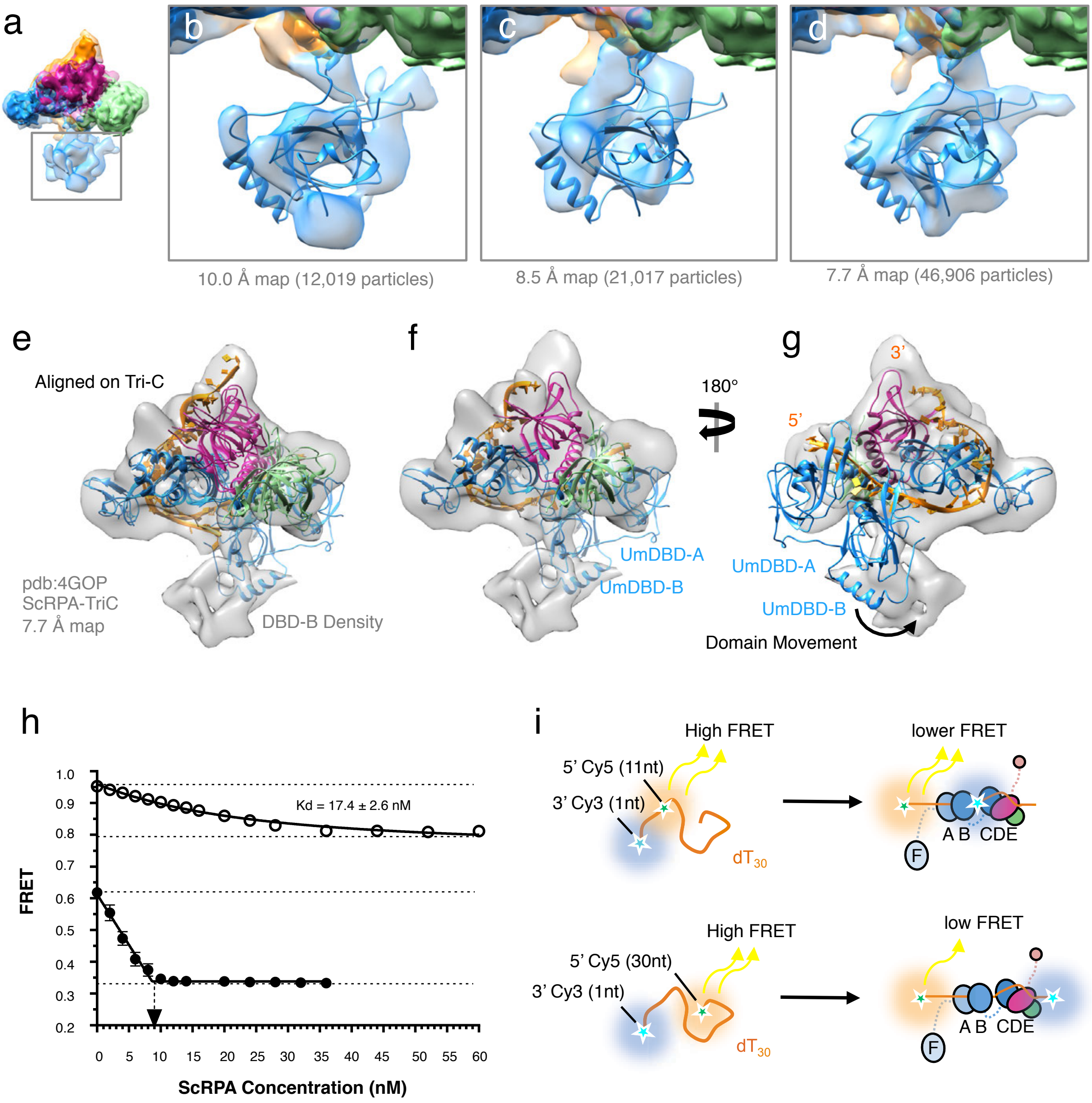

Supplementary Figure 5 – Conformational comparisons between RPA structures

(a) Reconstructions (from Fig. 2g) aligned on the Tri-C, colored as in Fig. 2, showing multiple locations of DNA-binding domain locations.

(b-d) Close up views of the additional DBD from individual reconstructions with a DBD-B homology model showing sufficient density to encompass an OB-fold. Resolutions and particle number is also shown for information.

(e) ScRPA Tri-C fitted into the 7.7 Å Tri-C with DBD-B together with the fungal RPA (pdb 4gop) alignment on ScRPA.

(f-g) Two views of the fitted fungal RPA structure (compact mode) showing different locations of DBD-A-DBD-B compared to the EM reconstructions

(h-i) Single molecule FRET analysis of ScRPA dT_{30} together with cartoons of the experiment (i) Black circles dT30 (1-30, also shown in Figure 4); open

circles dT30 (1-11) where the label position is given in parentheses. Data points for dT30 (1-11) are an average of three experiments. Based upon the crystal structure, positions 1-11 and 1-30 are located ~53 Å and 55 Å apart, respectively.

Supplementary Figure 6

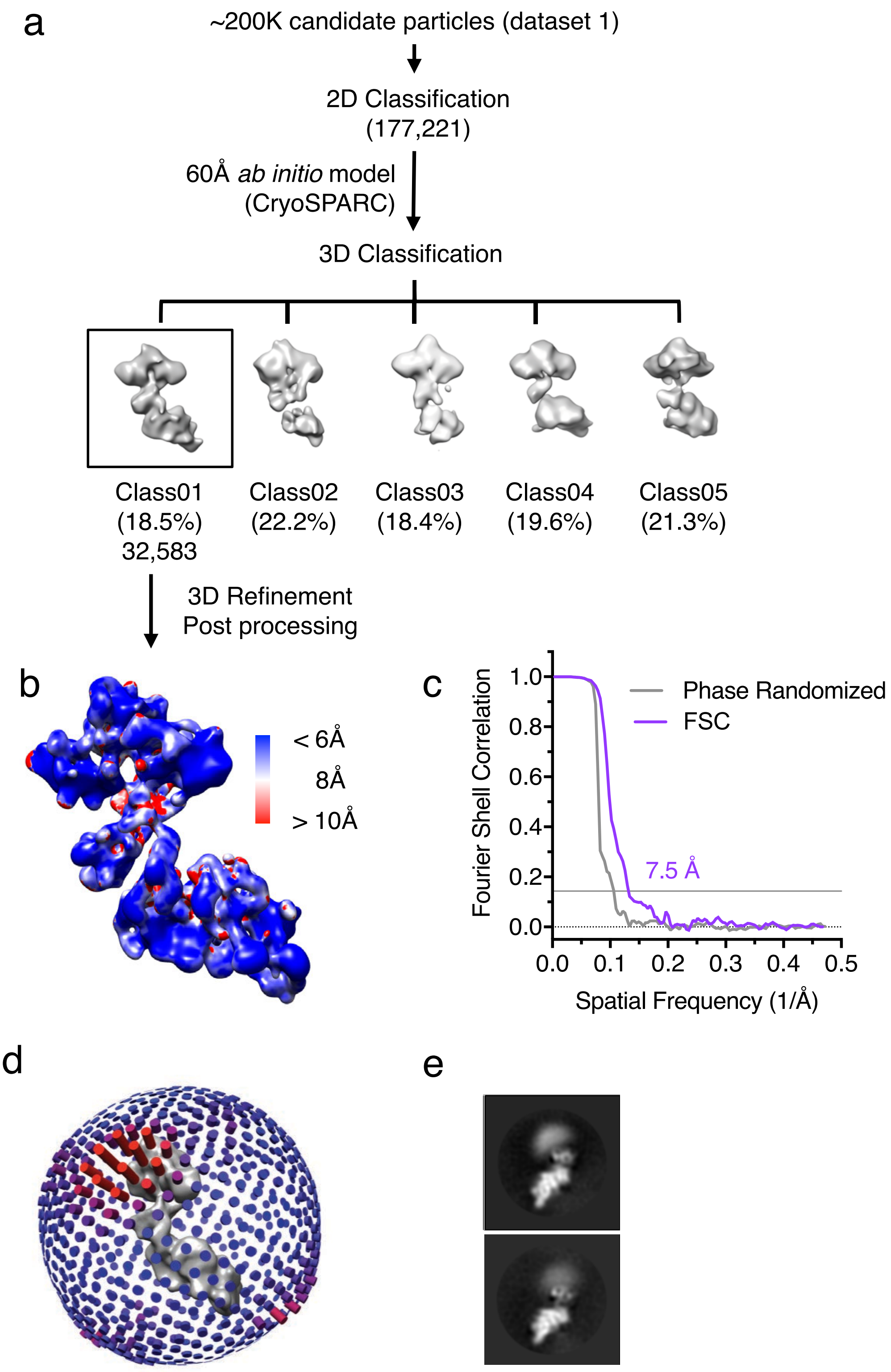

Supplementary Figure 6- Reconstruction scheme for the ScRPA-dT_{100} dimer

(a) 3D reconstruction schematic for ScRPA dimer showing processing of candidate particles that yielded a final 3D reconstruction.
(b) 7.5 Å dimer structure assessed by gold standard Fourier shell correlation (FSC).
(c) Gold-standard FSC curves from two independently refined half maps.
(d) Angular distribution of the RPA dimer structure.
(e) 2D-class averages of the dimer locally aligned on the 'bottom' Tri-C revealing the relative flexibility of this complex that ultimately precludes its high-resolution reconstruction.

Supplementary Figure 7

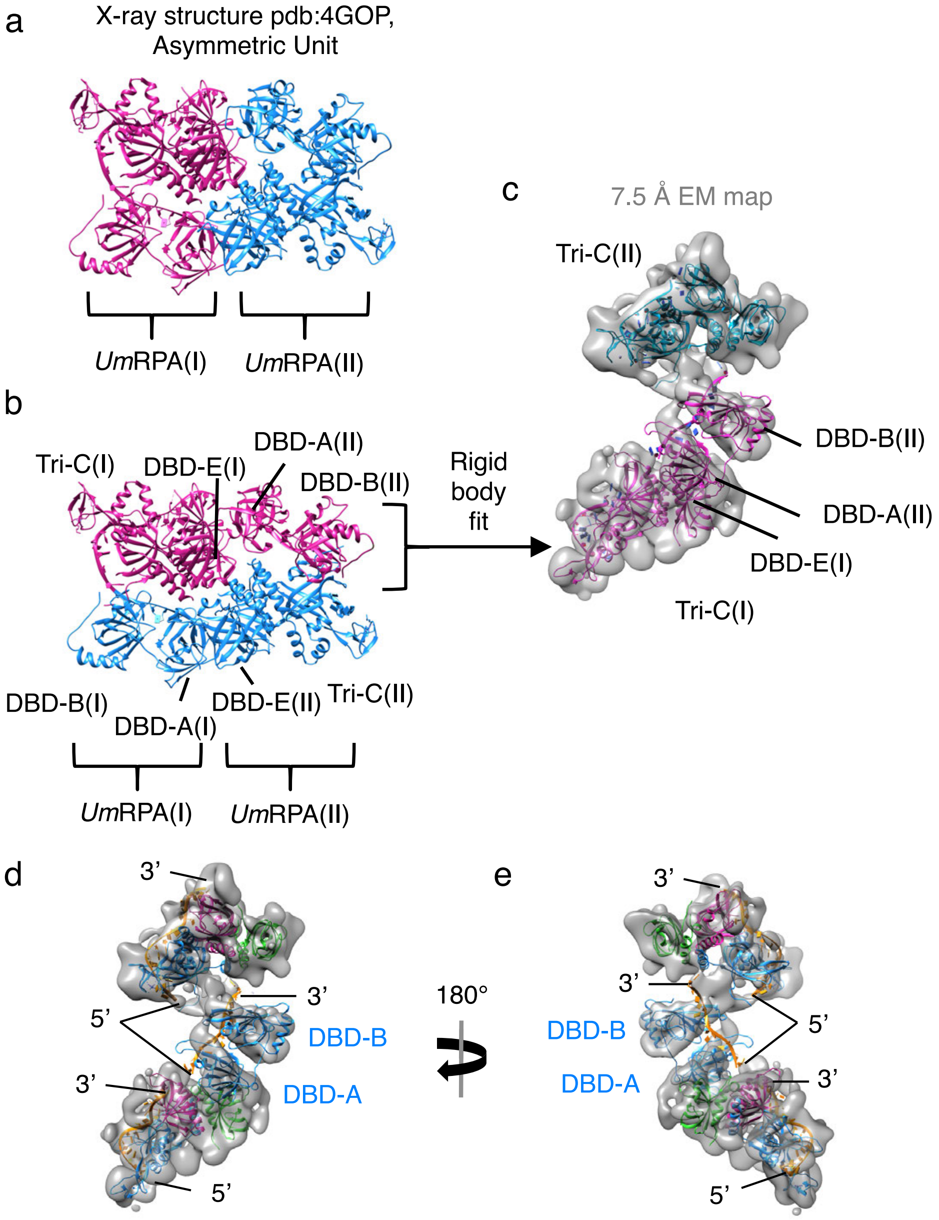

Supplementary Figure 7 – Analysis of the Dimer reconstruction
(a) Packing of the U. maydis RPA in the asymmetric unit (ASU) of its crystal structure (pdb 4GOP) with each RPA heterotrimer colored pink or blue.
(b) An interaction across the ASU showing the association between DBD-E and DBD-A of an adjacent molecule (domains labeled). The rigid body we use to dock into the lower half of the dimer EM map is colored pink.
(c) Coordinates of a trimerisation core of U. maydis RPA with an associated DBD-A/B tandem (from the adjacent molecule) in complex with ssDNA is taken directly from the asymmetric unit and docked into the lower half of the cryo-EM dimer density.

(d-e) Fitted U. maydis structure colored by subunit as in Fig 2. The ssDNA 5' and 3' ends are labeled to show the polarity and that a continuous ssDNA could traverse the entire dimer in an extended configuration.

Supplementary Figure 8

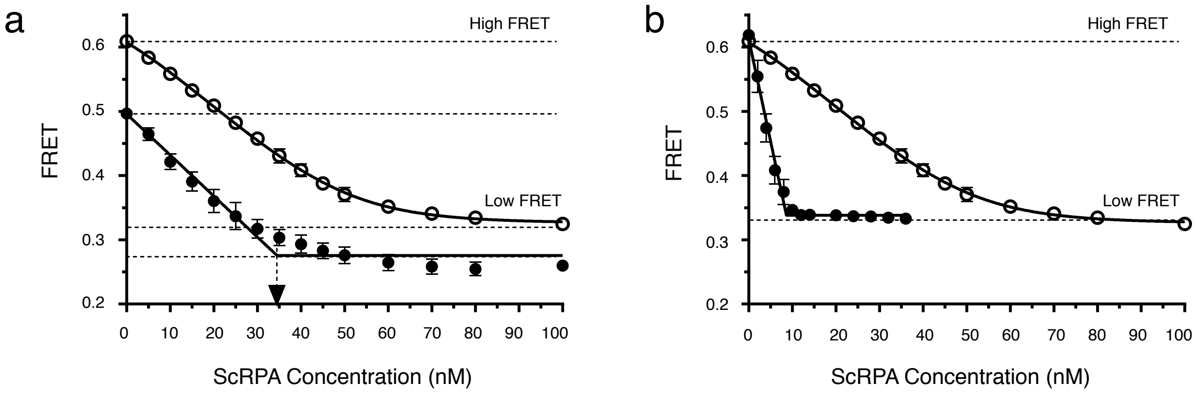

Supplementary Figure 8 – FRET-based analysis of the ssDNA configuration when bound by ScRPA

(a) scRPA binding to 10 nM of the dT_{90} (1-30) oligo (open circles) shows relatively low affinity binding (Kd 39.3 ± 0.9 nM), compared to dT90 (5-54) (black-filled circles), suggesting that scRPA prefers binding to the middle of the oligo over the 5' end. Both still show the same linearization of the ssDNA phenomenon. (b) Comparison between the dT_{30} (1-30) (black filled circles) and dT_{90}(1-30) (open circles). The same locations of the Cy5 dye at the 5'-end results in the same initial calculated FRET and the same final FRET at the titration end point. Experiments were repeated 3 times and data points are an average with standard deviation (SD).

Supplementary Figure 9

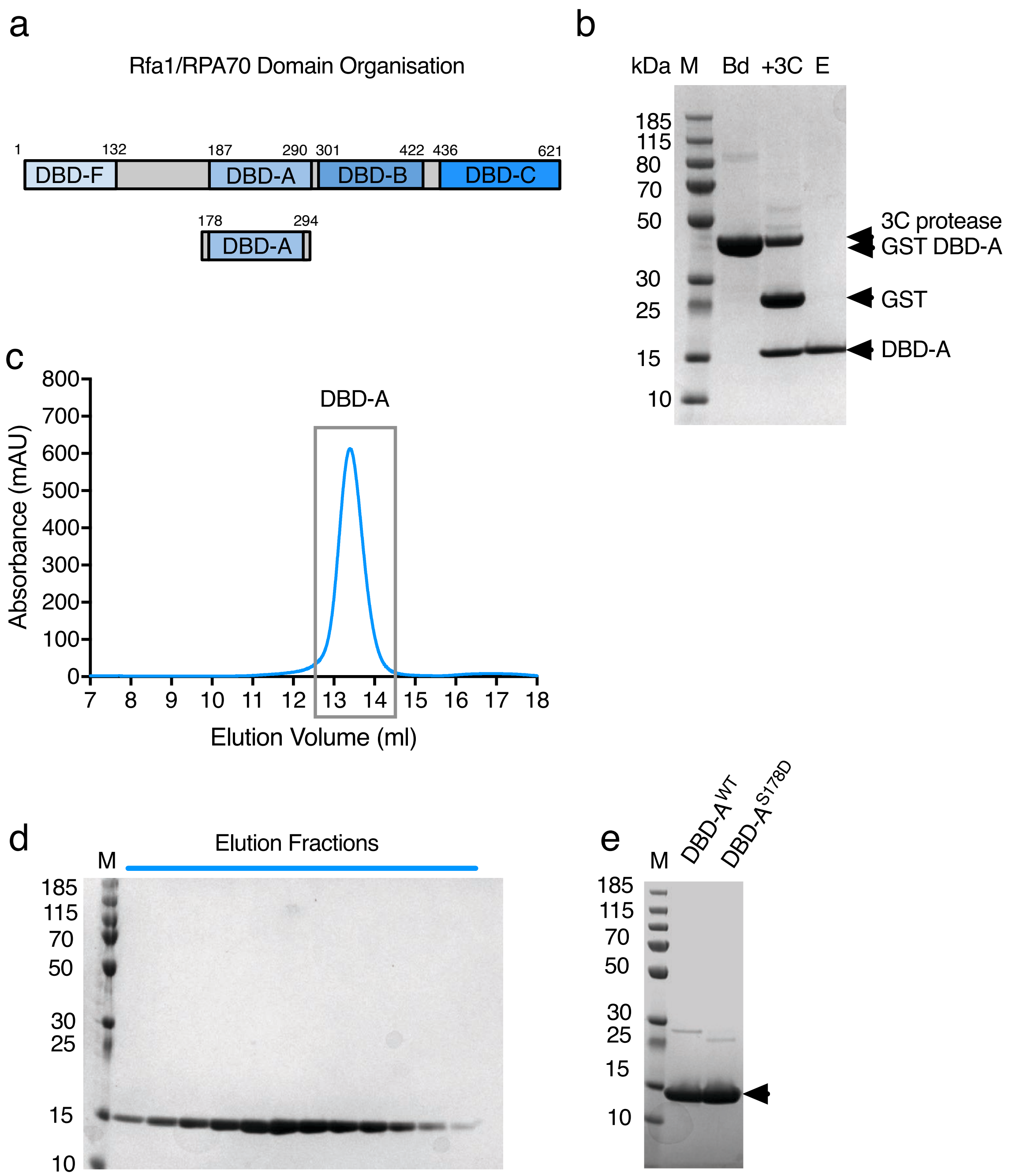

Supplementary Figure 9 – Purification of DBD-A

(a) Rfa1 domain schematic with domain boundaries used for construct design.
(b) SDS-PAGE showing purification scheme. GST-fused DBD-A was immobilized onto glutathione resin (lane, bd) and liberated by in situ 3C cleavage (lane, +3C) and the isolated DBD-A eluted (lane, E).
(c) Gel filtration profile of DBD-A using a superdex S75 (10/300).
(d) SDS-PAGE of the elution fractions from gel filtration.
(e) SDS-PAGE of purified and concentrated DBD-A and a DBD-A^{S178D} mutant.

Supplementary Figure 10

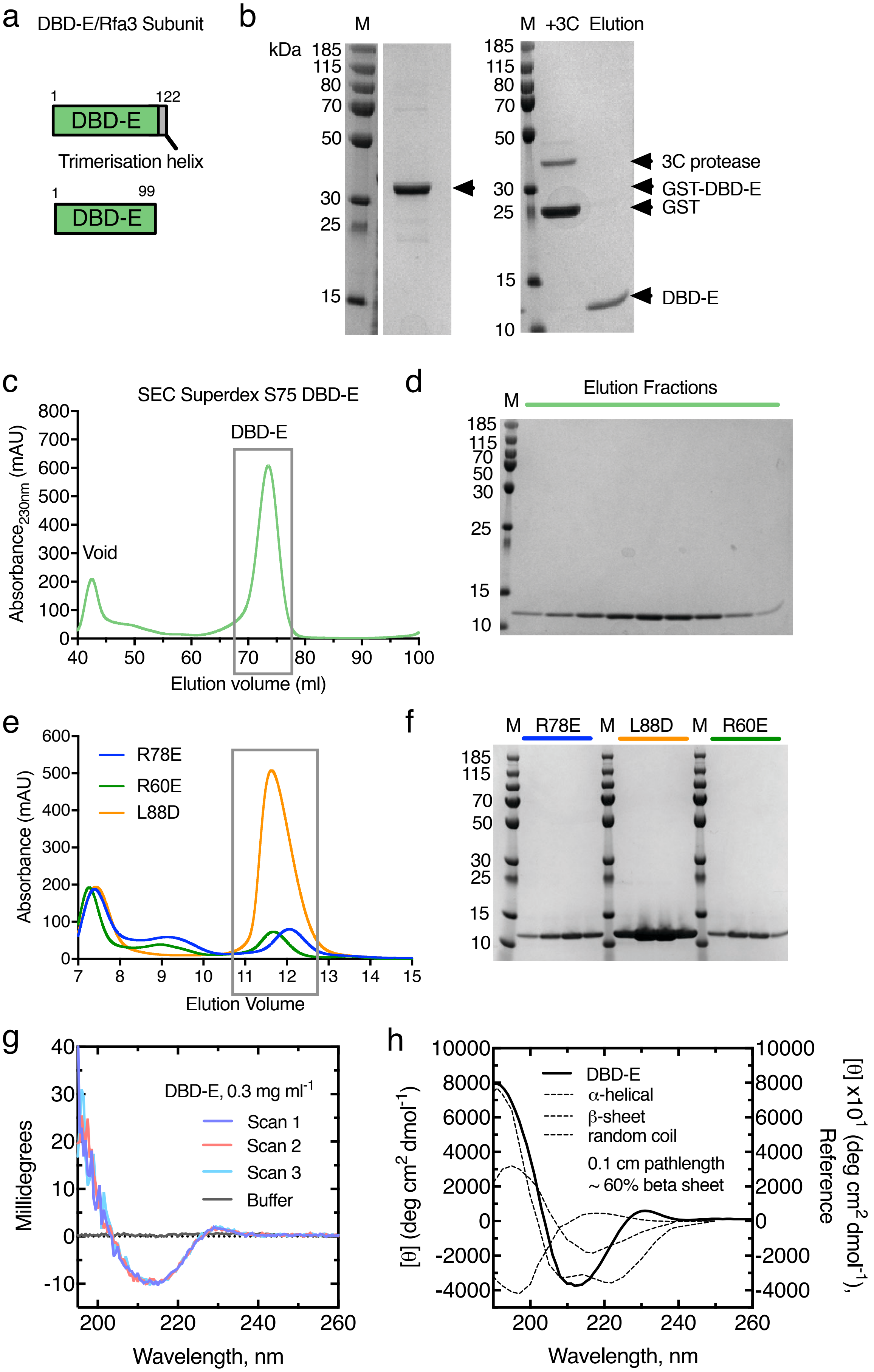

Supplementary Figure 10 – Purification of DBD-E

(a) DBD-E domain schematic with domain boundaries used for construct design.

(b) SDS-PAGE showing purification scheme. GST-fused DBD-E was immobilized onto glutathione resin and liberated by in situ 3C cleavage (lane, +3C) and the isolated DBD-A eluted (lane, Elution).

(c) Gel filtration profile of DBD-E using a superdex S75 (16/60).

(d) SDS-PAGE of the elution fractions from gel filtration.

(e-f) Gel filtration profiles of mutant DBD-E along with SDS-PAGE of purified and concentrated proteins.

(g-h) Circular Dichroic (CD) spectra of DBD-E demonstrating retention of secondary structure. Circular dichroic spectra were recorded at 0.1mg/ml at 25 ˚C on a Jasco J-815 circular dichroism spectrometer. Air and buffer scans were subtracted from the protein spectra. (h) The molar ellipticity was calculated using CAPITO [1] and suggests 60% beta sheet content consistent with an OB-fold. Reference traces from [2] are shown for comparison only.

Supplementary Figure 11

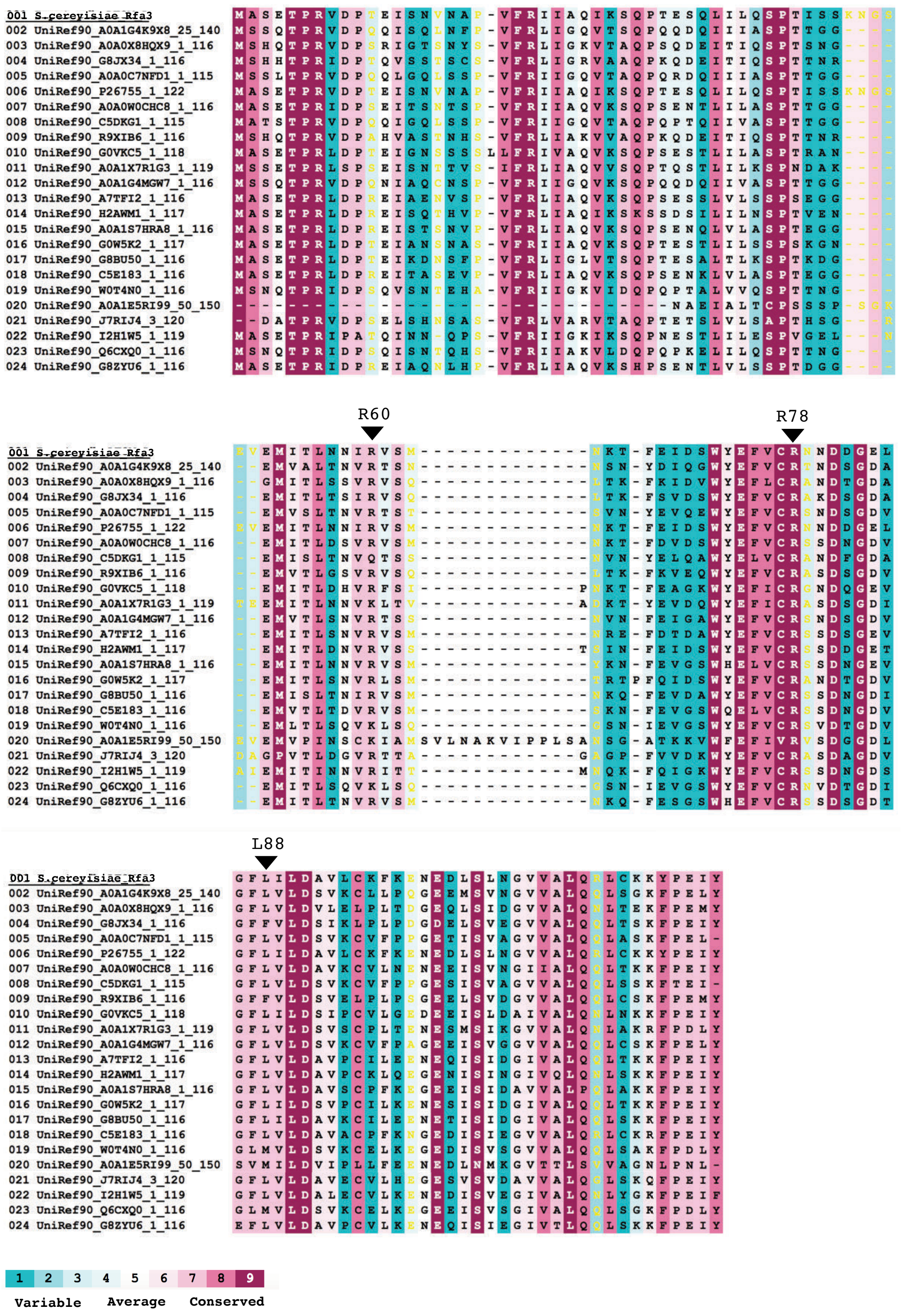

Supplementary Figure 11 – Multiple Sequence Alignment of Rfa3
Multiple amino acid sequence alignment of yeast Rfa3 using the Consurf server [3] for pairwise alignments and conservation scoring. A key is given for the color-coded conservation scores and the residues mutated in our investigation are shown and labeled.

Supplementary Figure 12

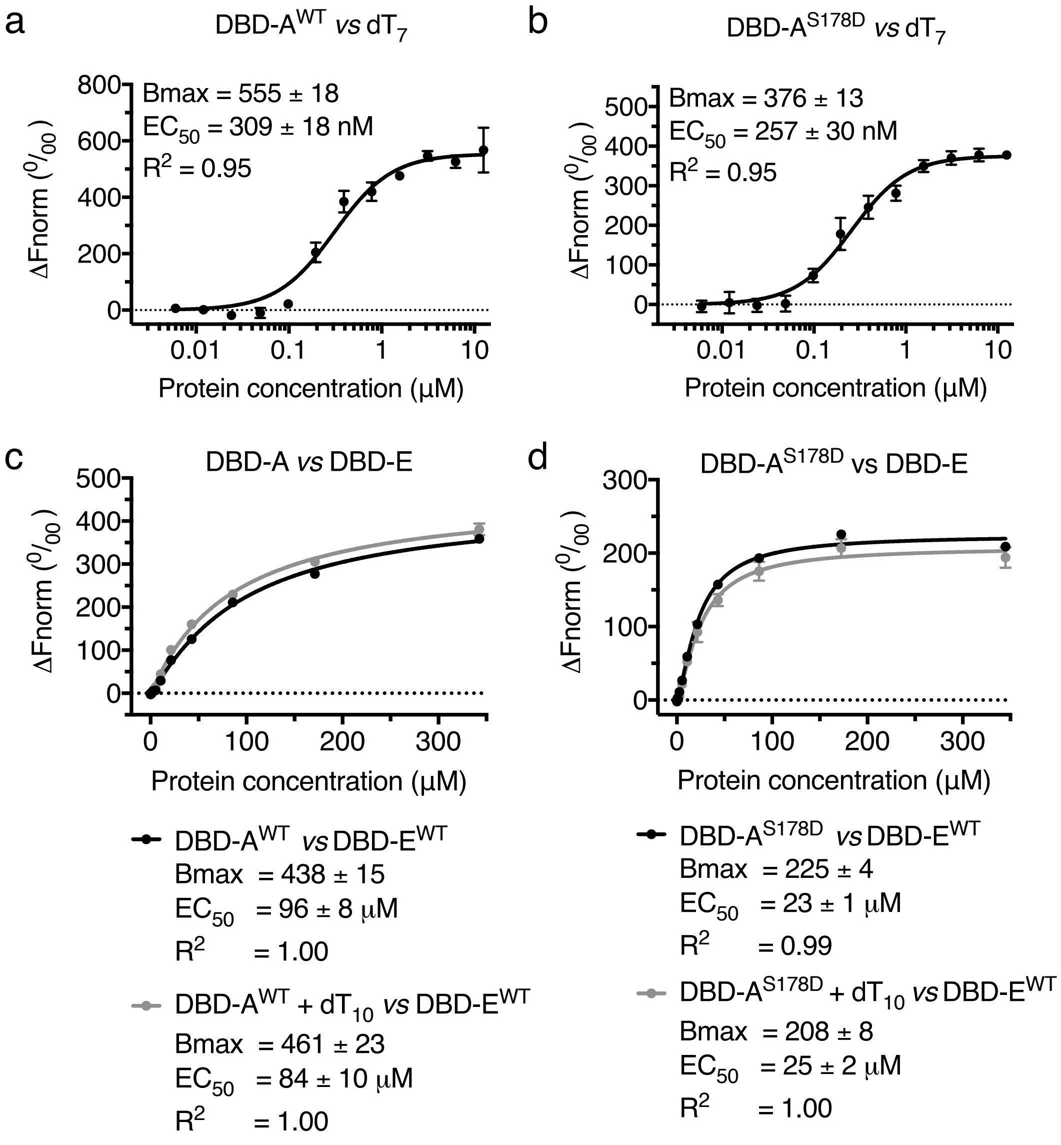

Supplementary Figure 12 – Interaction studies of DBD-A with ssDNA and the effect of ssDNA presence on its interaction with DBD-E

(a-b) Assessment of DBD-A and DBD-A^{S178D} domain interactions with ssDNA (Cy5-dT_7) using MST. The interaction between DBD-A and dT_7 produced protein induced fluorescence enhancement in a protein dependent manner. Normalized fluorescence is plotted against DBD-A concentration. The data were replicated 3 times and the average (with SEM) was fitted with the Hill equation in Prism7. (c-d) Assessment of DBD-A-DBD-E and DBD-A^{S178D}-DBD-E interactions in the presence of (grey trace) and absence (black trace) of excess ssDNA (dT_{10} at 2 µM in each binding reaction). Normalized fluorescence is plotted against DBD-A concentration. The data were replicated 3 times for DBD-A-DBD-E and twice in the presence of ssDNA. Data points are averages (with SEM) and fitted with the Hill equation in

Prism7. In all cases, the calculated Bmax, EC_{50} and R^2 are shown for the fitting together with fitting error.

Supplementary Figure 13

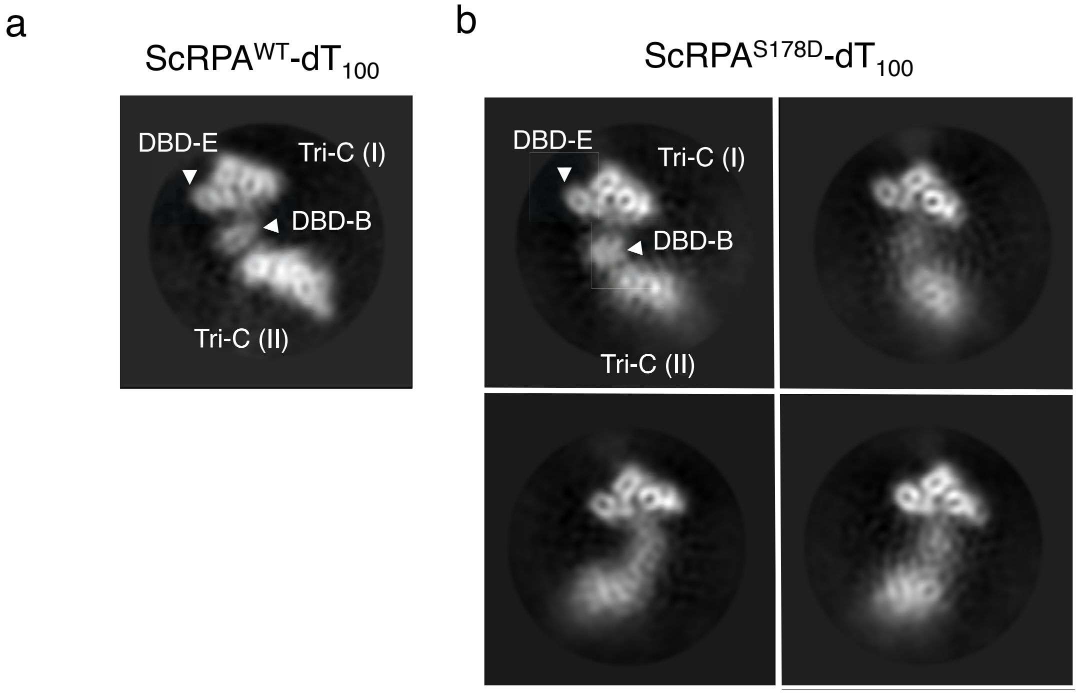

Supplementary Figure 13 – CryoEM 2D image analysis of ScRPA^{S178D}-dT$_{100}$

(a) 2D class averages of ScRPA-dT100 showing two well-ordered Tri-C regions. DBD-E and DBD-B are labeled for orientation purposes.

(b) 2D class averages of ScRPA^{S178D}-dT100 showing the top Tri-C in approximately the same orientation as (a) but shows diffuse density regions suggesting the an increased mobility/flexibility between connecting RPAs.

Supplementary Figure 14

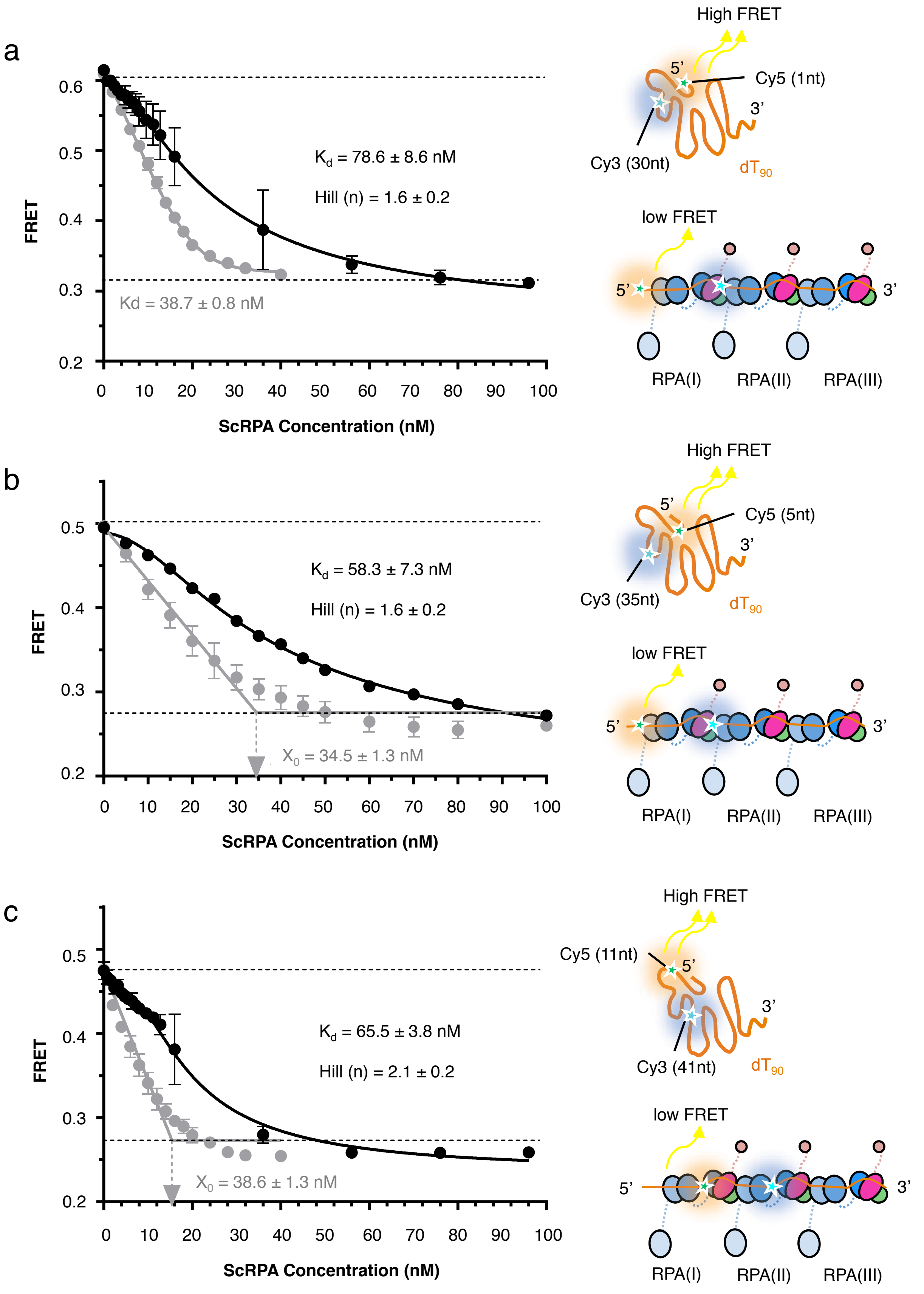

Supplementary Figure 14 – FRET-based analysis of ssDNA configuration when bound by ScRPA^{S178D}

(a-c) Indicated concentrations of ScRPA^{S178D} (black circles) were added to the solution containing 10 nM dT_{30} oligo decorated with the Cy3 dye (FRET donor) towards the 3' and the Cy5 dye (FRET acceptor) towards the 5' end. For comparison, experiments on the same substrate with ScRPA^{WT} are shown in grey and where possible Kd and X_0 values provided (see Fig. 4). FRET-labelled oligonucleotides produce a high FRET signal with protein binding and ssDNA straightening resulting in a reduction of signal. Cartoons depicting the high (unbound) and low (bound) FRET states are provided. The FRET labels are 30nt apart and the nucleotide positions are given in parentheses. The mutant requires a higher concentration of protein to reach saturation and the data can be fitted with the Hill equation to provide a Kd and a Hill coefficients, which are given. Experiments were repeated 3 times and data points are an average with standard deviation (SD).

Supplementary Figure 15

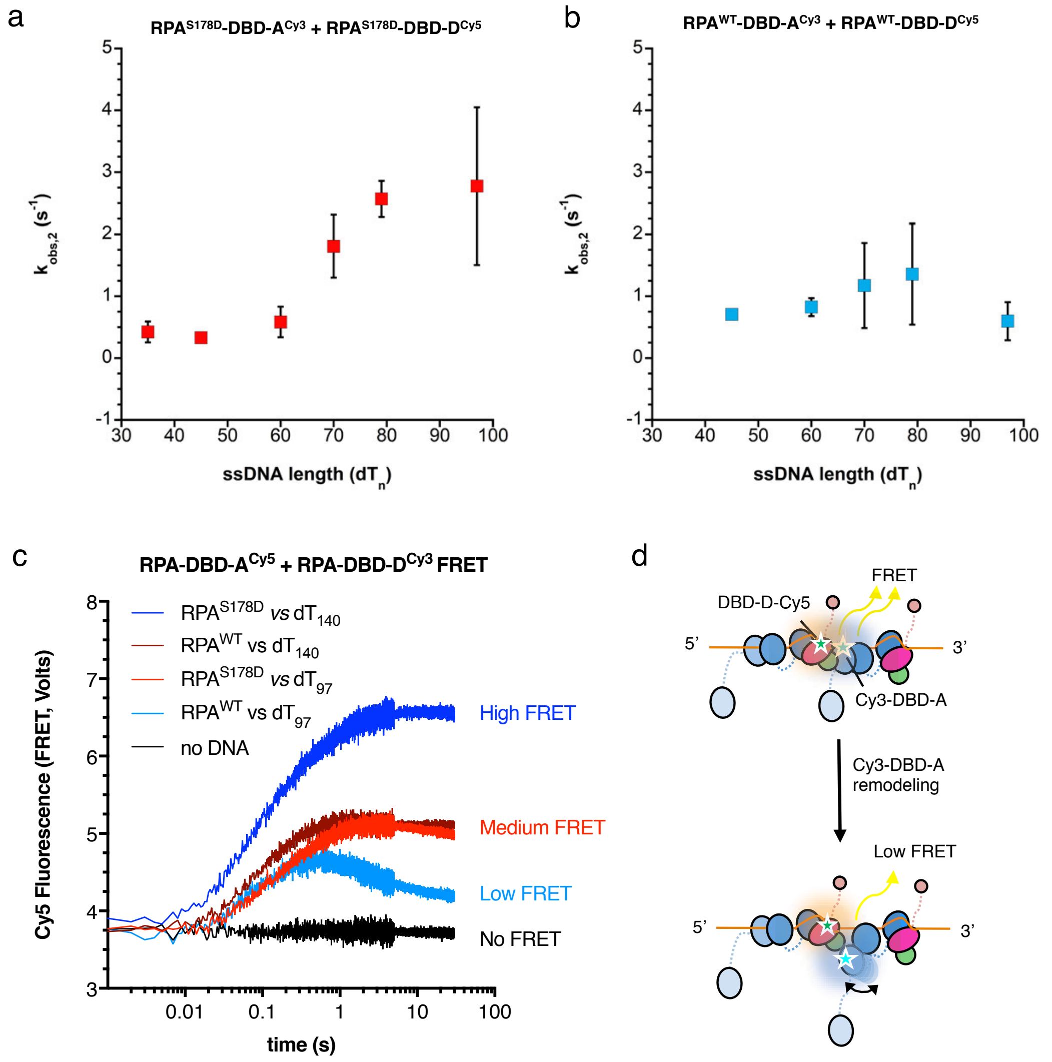

Supplementary Figure 15 – FRET-based assessment of RPA-RPA

(a-b) Second rates derived from the fitting of FRET data in Fig 6. $k_{obs,2}$ is plotted as a function of ssDNA length (nucleotides). Data points are averages of 3 experiments and are shown with SEM.

(c) Stopped flow fluorescence measurements of RPA^{WT}-DBD-D-Cy5 with RPA^{WT}-DBD-A-Cy3 (blue trace) and RPA^{S178D}-DDB-D-Cy5 with RPA^{S178D}-DBD-A-Cy3 (red trace), with the protein concentration in excess over the ssDNA substrate (dT_{97}). FRET signal signatures indicate that for RPA^{WT} its DBD-A can be remodeled over time due to binding site competition, whereas RPA^{S178D} is more stable.

(d) Cartoon models of RPA configurations that lead to the different FRET signatures.

Supplementary Figure 16

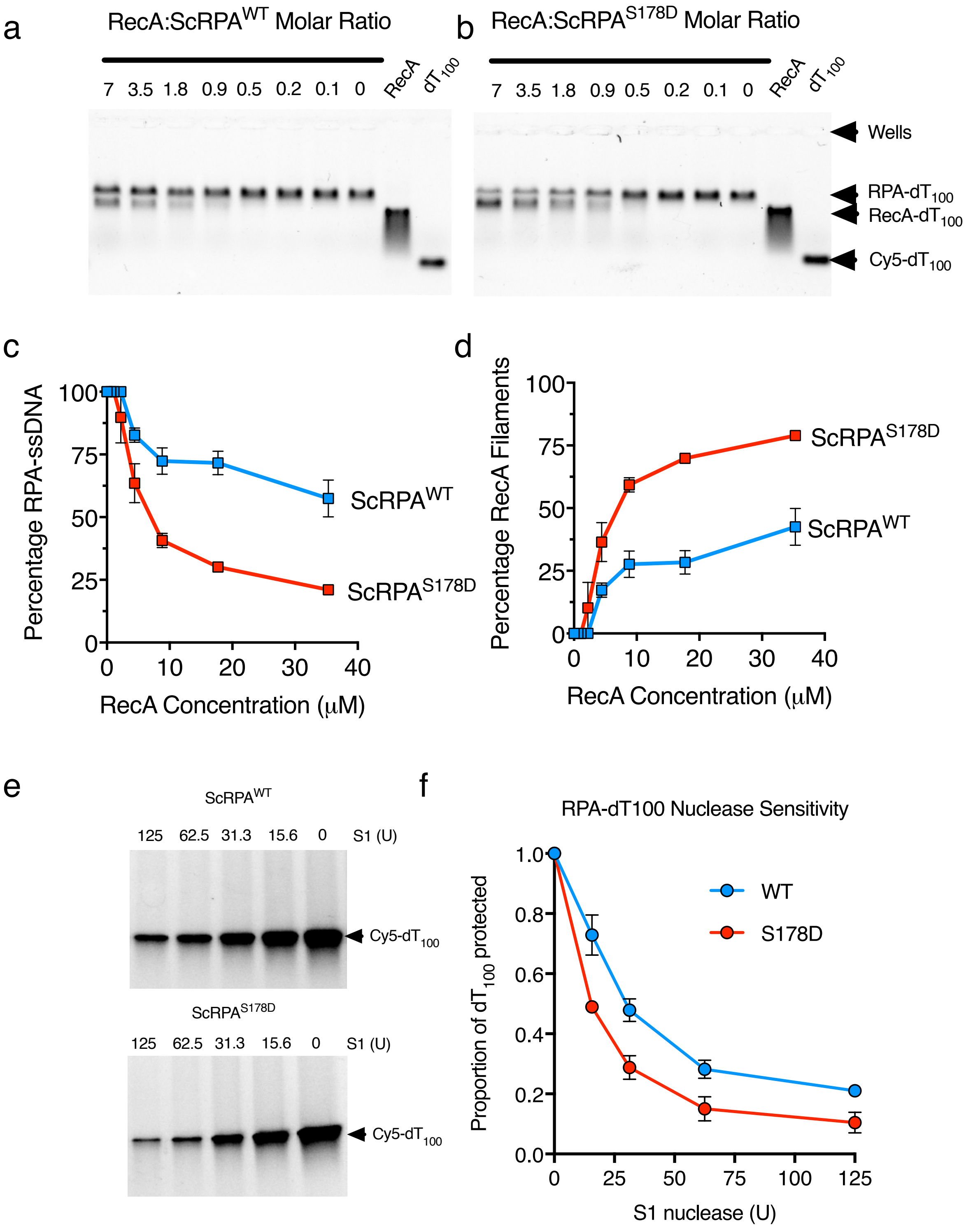

Supplementary Figure 16 – RecA and S1 nuclease sensitivity assay

(a-d) RecA exchange assay of (a) ScRPA^{WT}-dT$_{100}$ and (b) ScRPA^{S178D}-dT$_{100}$ nucleoprotein complexes resolved by EMSA. The molar ratio between RecA and RPA are given for each lane. The relative proportions of band intensities for species corresponding to either (c) RPA-dT100 complexes or (d) RecA-dT100 complexes are plotted for WT and S178D mutant against RecA concentration.

(e) S1 nuclease sensitivity assay. ScRPA-Cy5-dT$_{100}$ complexes were incubated with S1 nuclease (NEB) for 30 minutes, the reaction halted by SDS-PAGE loading buffer, and the ssDNA resolved on 4-20% polyacrylamide gel under denaturing conditions.

(f) Band intensities of the Cy5-dT100 were expressed as a fraction of the untreated sample and plotted against amount of S1 nuclease (units).

IN all cases, data points are an average of three experiments and are shown with SEM.

Supplementary Table 1: Oligonucleotides used in this study

**Name**	**Primer Sequence** Lowercase denotes InFusion overlaps, underlined sequence indicates mutagenesis site.	**Protein Product**
Rfa1_DBD-A_f	aagttctgtttcagggcccgGATGAAAGCAATGTGCCGAAAACCCAC	DBD-A (178-294)
Rfa1_DBD-A_r	atggtctagaaagctttaCGGTTCTTGTTTCAGCGTAATAAAGTTGGCG	DBD-A (178-294)
Rfa3_OB_f	aagttctgtttcagggcccgATGGCAAGCGAAACACCGCG	DBD-E (1-99)
Rfa3_OB_r	atggtctagaaagctttaATTTTCTTTGAATTTACACAGAACGGCATCCAGAATC	DBD-E (1-99)
Rfa1_S178D_f	GCAAATGAAAATCCGAATGATCAGAAAACC	$\text{RPA}^{\text{S178D}}$
Rfa1_S178D_r	GGTTTTCTGATCATTCGGATTTTCATTTGC	$\text{RPA}^{\text{S178D}}$
Rfa3_R78E_f	GAATTTGTGTGCGAAAATAATGATGATGG	$\text{DBD-E}^{\text{R78E}}$
Rfa3_R78E_r	CCATCATCATTATTTTCGCACACAAATTC	$\text{DBD-E}^{\text{R78E}}$
Rfa3_R60E_f	CCCTGAATAACATTGAAGTGAGCATGAAC	$\text{DBD-E}^{\text{R60E}}$
Rfa3_R60E_r	GTTCATGCTCACTTCAATGTTATTCAGGG	$\text{DBD-E}^{\text{R60E}}$
Rfa3_L88D_f	GGTGAACTGGGTTTTGATATTCTGGATGCC	$\text{DBD-E}^{\text{L88D}}$
Rfa3_L88D_r	GGCATCCAGAATATCAAAACCCAGTTCACC	$\text{DBD-E}^{\text{L88D}}$
**Name**	**Substrates used in the FRET-based analysis**	
dT_{30}	5'-/Cy5/TTT TTT TTT TTT TTT TTT TTT TTT TTT TTT/Cy3/-3'	
dT_{90}(1-30)	5'-/Cy5/TTT TTT TTT TTT TTT TTT TTT TTT TTT TT/Cy3/T TTT TTT TTT TTT TTT TTT TTT TTT TTT TTT TTT TTT TTT TTT TTT TTT TTT TTT TTT TTT -3'	
dT_{90}(5-35)	5'-TTT T/Cy5/TT TTT TTT TTT TTT TTT TTT TTT TTT TTT T/Cy3/TT TTT TTT TTT TTT TTT TTT TTT TTT TTT TTT TTT TTT TTT TTT TTT TTT TTT TTT -3'	
dT_{90}(11-41)	5'-TTT TTT TTT T/Cy5/TT TTT TTT TTT TTT TTT TTT TTT TTT TTT T/Cy3/TT TTT TTT TTT TTT TTT TTT TTT TTT TTT TTT TTT TTT TTT TTT TTT TTT TTT -3'	

Supplementary References

1. Wiedemann, C., Bellstedt, P. & Görlach, M. CAPITO--a web server-based analysis and plotting tool for circular dichroism data. *Bioinformatics* **29,** 1750–1757 (2013).

2. Greenfield, N. J. Using circular dichroism spectra to estimate protein secondary structure. *Nat Protoc* **1,** 2876–2890 (2006).

3. Ashkenazy, H. *et al.* ConSurf 2016: an improved methodology to estimate and visualize evolutionary conservation in macromolecules. *Nucleic Acids Res* **44,** W344–50 (2016).
